# The Angio-Fibrotic Switch of VEGF and CTGF in Proliferative Diabetic Retinopathy

**DOI:** 10.1371/journal.pone.0002675

**Published:** 2008-07-16

**Authors:** Esther J. Kuiper, Frans A. Van Nieuwenhoven, Marc D. de Smet, Jan C. van Meurs, Michael W. Tanck, Noelynn Oliver, Ingeborg Klaassen, Cornelis J. F. Van Noorden, Roel Goldschmeding, Reinier O. Schlingemann

**Affiliations:** 1 Ocular Angiogenesis Group, Departments of Ophthalmology and Cell Biology and Histology, Academic Medical Center, University of Amsterdam, Amsterdam, The Netherlands; 2 Department of Pathology, Academic Medical Centre of Utrecht, Utrecht, The Netherlands; 3 Department of Ophthalmology, Rotterdam Eye Hospital, Rotterdam, The Netherlands; 4 Department of Clinical Epidemiological Statistics, Academic Medical Center, University of Amsterdam, Amsterdam, The Netherlands; 5 FibroGen Inc, San Francisco, California, United States of America; University of Sydney, Australia

## Abstract

**Background:**

In proliferative diabetic retinopathy (PDR), vascular endothelial growth factor (VEGF) and connective tissue growth factor (CTGF) cause blindness by neovascularization and subsequent fibrosis, but their relative contribution to both processes is unknown. We hypothesize that the balance between levels of pro-angiogenic VEGF and pro-fibrotic CTGF regulates angiogenesis, the angio-fibrotic switch, and the resulting fibrosis and scarring.

**Methods/Principal Findings:**

VEGF and CTGF were measured by ELISA in 68 vitreous samples of patients with proliferative DR (PDR, N = 32), macular hole (N = 13) or macular pucker (N = 23) and were related to clinical data, including degree of intra-ocular neovascularization and fibrosis. In addition, clinical cases of PDR (n = 4) were studied before and after pan-retinal photocoagulation and intra-vitreal injections with bevacizumab, an antibody against VEGF. Neovascularization and fibrosis in various degrees occurred almost exclusively in PDR patients. In PDR patients, vitreous CTGF levels were significantly associated with degree of fibrosis and with VEGF levels, but not with neovascularization, whereas VEGF levels were associated only with neovascularization. The ratio of CTGF and VEGF was the strongest predictor of degree of fibrosis. As predicted by these findings, patients with PDR demonstrated a temporary increase in intra-ocular fibrosis after anti-VEGF treatment or laser treatment.

**Conclusions/Significance:**

CTGF is primarily a pro-fibrotic factor in the eye, and a shift in the balance between CTGF and VEGF is associated with the switch from angiogenesis to fibrosis in proliferative retinopathy.

## Introduction

Blindness from proliferative diabetic retinopathy (PDR) is caused by angiogenesis and fibrosis in the vitreous cavity of the eye [1]–[4]. PDR is a wound healing-like response in which neovascularization is accompanied by influx of inflammatory cells and development of myofibroblasts. This progresses to a fibrotic phase with fibrovascular contraction causing haemorrhages, retinal detachment and inevitable blindness. Several growth factors have been shown to play a role in PDR, *e.g.* vascular endothelial growth factor-A (VEGF), transforming growth factor-ß, hepatocyte growth factor, platelet-derived growth factor, and the pro-fibrotic connective tissue growth factor (CTGF) [5], [6]. VEGF is considered to be the primary angiogenesis factor in this sequence of events [7]–[9]. In contrast, the causal factors of fibrosis and scarring and the regulation of the transition from angiogenesis to the fibrotic phase of PDR, which we propose to call the angio-fibrotic switch, remain largely unknown. We have recently shown that vitreous levels of CTGF correlate strongly with degree of fibrosis in vitreo-retinal disorders [10]. In other organs than the eye, CTGF has a similar role and has been identified as a potential therapeutic target for inhibition of pathological fibrosis [11]–[15].

Interestingly, CTGF is upregulated by VEGF [16]–[18] and inhibits VEGF-induced angiogenesis by formation of VEGF-CTGF complexes [19], [20], suggesting a negative feedback function of CTGF on VEGF-induced angiogenesis. In contrast, recombinant CTGF has been shown to cause angiogenesis in the cornea and in other in vivo models in addition to its pro-fibrotic properties [21], [22]. Other findings, such as co-localization of CTGF and VEGF in human subretinal neovascularization [23], and increased CTGF levels in vitreous of PDR patients with active neovascularization [24], also suggested that CTGF has a causal role in ocular neovascularization. However, we have recently challenged this concept by showing that in CTGF knock-out mice, angiogenesis is not impaired in several experimental models of neovascularization [25].

Based on these findings we propose a novel concept. We hypothesize that CTGF is a primary causal factor in sight-threatening fibrosis in the eye, and that an equilibrium shift in the balance between VEGF and CTGF levels in vitreous regulates the switch from the angiogenic phase to fibrosis in conditions such as PDR. Therefore, we investigated vitreous VEGF and CTGF levels in correlation with degree of fibrosis and neovascularization in a series of patients with PDR and other vitreoretinal disorders, and studied the effect of anti-VEGF treatment on the course of PDR in human patients.

## Methods

### Patients

We investigated 68 vitreous samples of patients with PDR (N = 32), macular hole (N = 13) and idiopathic macular pucker (N = 23), who were operated by pars plana vitrectomy. The study was conducted according to the Declaration of Helsinki and approved by the institutional review board of the Academic Medical Center at the University of Amsterdam. Informed written consent was obtained from each patient.

Clinical data, which allowed grading of fibrosis, activity of neovascularization, degree of haemorrhage, and presence and type of diabetes, were obtained from the pre-operative ophthalmic and ultrasound examinations, the patient files and from per-operative observations, using a standardized form. Fibrosis was graded as 0 when there was no fibrosis, as 1 when there were a few pre-retinal membranes (as limited as in macular pucker), as 2 when white pre-retinal fibrotic membranes with limited extension into the vitreous were present, and as 3 when abundant white membranes reaching into the vitreous body were observed. Neovascularization was graded as 0 when absent, as 1 (quiescent) when only non-perfused vessels were present, and as 2 (active) when there were perfused preretinal capillaries [26]. Degree of haemorrhage was graded as 0 when all media were clear and all fundus details were visible, as 1 when media were a little clouded but the fundus could still be examined, as 2 when the disk was obscured by haemorrhage, and as 3 when fundus details could not be obtained.

In addition, we investigated retrospectively, after informed consent, the clinical records of 4 patients with PDR and one patient with proliferative retinopathy as a complication of branch retinal vein occlusion, who were treated with either pan-retinal photocoagulation (2 cases of PDR and the case of branch retinal vein occlusion) or with intravitreal injections with bevacizumab, an anti-VEGF agent, followed by pan-retinal laser treatment (2 cases of PDR).

### Sample collection

Undiluted vitreous samples (0.5–1 ml) were obtained by using a vitrectome at the start of a classic three-port pars plana vitrectomy with the infusion line in position but not opened. The vitreous was transferred to sterile Eppendorf tubes and immediately frozen in dry ice. The samples were kept at −80°C until assayed.

### Measurement of CTGF and VEGF levels by enzyme-linked immunosorbent assays (ELISA)

After thawing, vitreous samples were centrifuged at 14.000 rpm for 15 minutes at 4°C, and supernatant was collected. Concentrations of CTGF were measured in samples by means of sandwich ELISAs, using two distinct monoclonal antibodies specifically recognizing the N-terminal part of the CTGF protein (FibroGen, San Francisco CA, USA) as described previously [10]. Purified recombinant human CTGF (FibroGen) was used as standard. Concentrations of VEGF_165_ were determined by means of sandwich ELISA according to the manufacturer's protocol (R&D Systems, Minneapolis MS, USA). All ELISAs were carried out in duplicate.

### Statistical analysis

Both CTGF and VEGF levels were tested for normal distribution using the Shapiro-Wilk test (W>0.90). VEGF levels in vitreous showed a right skewed distribution and were log_10_ transformed to obtain a normal distribution. Associations between gender, age, diabetes type, degree of neovascularization, degree of haemorrhage and degree of fibrosis were assessed by ANOVA with correction for age and gender (included as covariates) where appropriate, and for unequal variances due to inter-assay variability. Least square means were estimated based on the model and, in case of an overall significant difference, compared using a post-hoc test. The correlation between CTGF and log_10_(VEGF) is expressed as Spearman correlation coefficient. In addition, univariate and multiple ordinal logistic regression analyses were performed with degree of fibrosis or neovascularization activity as dependent (outcome) variables. These effects were expressed as odds ratios with a 95% confidence interval. A two-tailed p-value <0.05 was considered statistically significant. All analyses were carried out using SAS (Version 9) software (SAS Institute, Cary NC, USA).

## Results

### Analysis of all patients

The research variables and characteristics of the 68 patients studied are shown in Table 1. There was a significant difference in age between the patients diagnosed with a macular hole or macular pucker and patients with PDR (66.4 versus 54.2, respectively; p = 0.001). Mean vitreous levels of CTGF and log_10_(VEGF) were significantly higher in patients with PDR than in patients diagnosed with a macular hole or macular pucker (p<0.001 for both growth factors, corrected for age (covariate)).

**Table 1:** Characteristics of the 68 patients included in the study.

Patient characteristics (n = 68)	Subcategory	Macular hole (n = 13)	PDR (n = 32)
		Macular pucker (n = 23)	
Age (mean±SD)		66·4±14·6	54·2±12·7
Gender	Male	15	15
	Female	21	17
Patients with diabetes	Total	0	32
	Type I		16
	Type II		16
Degree of neovascularization	No neovascularization (0)	36	2
	Quiescent neovascularization (1)		11
	Active neovascularization (2)		19
Degree of haemorrhage	No haemorrhage (0)	36	11
	Media little clouded (1)		4
	Disc obscured (2)		11
	No fundus detail visible (3)		6
Degree of fibrosis	No fibrosis (0)	13	0
	Only a few pre-retinal membranes (1)	23	2
	Some proliferative membranes (2)		17
	Abundant proliferative membranes (3)		13
			
CTGF (geom. mean, 95% CI)		13·0 ng/ml [10.8–15.3]	24.1 ng/ml [20.5–27.7]
VEGF (geom. mean, 95% CI)		123 pg/ml [95–159]	776 pg/ml [566–1065]
Ratio CTGF/log_10_(VEGF)		3.42 [2.7–4.2]	6.2 [5.3–7.1]

Abbreviations: PDR, proliferative diabetic retinopathy; SD, standard deviation; VEGF, vascular endothelial growth factor; CTGF, connective tissue growth factor.

**Figure 1:**
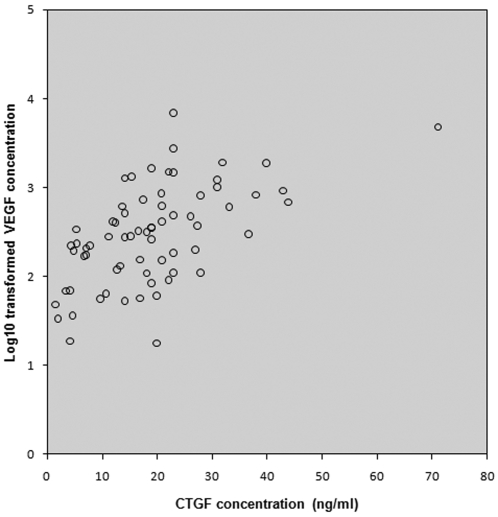
Correlation between the levels of CTGF and log_10_(VEGF) in the vitreous of all 68 patients. A significant (p = 0.01) Spearman's rank correlation (ρ = 0.4) within all samples was found.

Univariate analysis of all 68 patients showed that CTGF and log_10_(VEGF) levels in vitreous correlated strongly (p<0.001). A scatter plot of the correlation between CTGF levels and log_10_(VEGF) levels is shown (Figure 1). Univariate analysis of all 68 patients also showed that levels of both growth factors were associated with diabetes-associated variables (i.e. the presence of diabetes, degree of neovascularization, fibrosis and haemorrhage; all p<0.001). CTGF and log_10_(VEGF) levels did not differ between genders. Log_10_(VEGF) levels were not associated with age, whereas CTGF levels were (p = 0.03). Multivariate analysis showed that mean CTGF levels were only associated with degree of fibrosis (p<0.001), whereas log_10_(VEGF) levels were only significantly associated with degree of neovascularization (p<0.001).

### Analysis of patients with PDR

**Figure 2:**
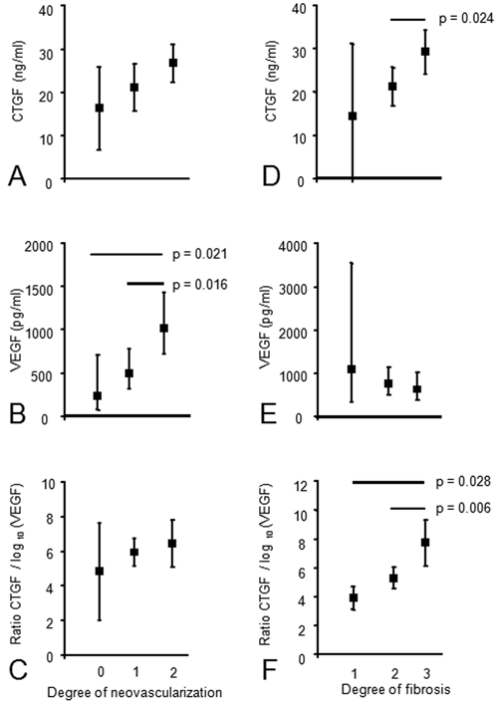
Mean levels of CTFG (A, D), geometric mean levels of VEGF (B, E), and mean ratio CTGF/log_10_(VEGF) (C, F) in relation with degree of neovascularization (A–C) and degree of fibrosis (D–F) in the vitreous of 32 PDR patients. Vertical bars represent 95% confidence intervals. Significant differences between groups are indicated.

Most parameters which correlated with VEGF and CTGF levels were associated with diabetes (i.e. neovascularization, haemorrhage and fibrosis) in the group of 68 patients. However, the 13 macular hole patients and 23 macular pucker patients did not suffer from diabetes (Table 1). Therefore, to exclude this confounder, we repeated the statistical analysis on the data of the 32 patients with PDR who all had diabetes. Similar to the entire patient population, CTGF levels in the vitreous of patients with PDR were significantly associated with degree of fibrosis (p = 0.042), and not significantly with degree of neovascularization (p = 0.072), whereas log_10_(VEGF) levels were associated with degree of neovascularization (p = 0.011), and not with degree of fibrosis (p = 0.665) (Figure 1). We also evaluated the association between the ratio CTGF/log_10_(VEGF) and degree of fibrosis or degree of neovascularization, respectively. The ratio CTGF/log_10_(VEGF) was associated with degree of fibrosis (p = 0.009), but not with degree of neovascularization (p = 0.635) (Figure 2).

The Spearman correlation coefficient for CTGF and log_10_(VEGF) levels was 0.4 (p = 0.01). Age, gender, degree of haemorrhage did not show an association with CTGF or log_10_(VEGF) levels (p>0.05).

In a separate analysis, an ordinal logistic regression model with degree of fibrosis as dependent (outcome) variable was used to sort out the strongest predictive variable for fibrosis. Univariate ordinal logistic regression model analysis (Table 2) showed that vitreous levels of CTGF and CTGF/log_10_(VEGF) ratios were associated with fibrosis. The association was not found between log_10_(VEGF) levels and degree of fibrosis but it was found between log_10_(VEGF) levels and degree of neovascularization.

**Table 2:** Odds ratio (OR) and 95% confidence interval (95%CI) of the univariate ordinal logistic regression models with degree of neovascularization or fibrosis as outcome in the PDR population (N = 32), respectively.

Variable	Contrast	Degree of neovascularization			Degree of fibrosis		
		OR	[95% CI]	P-value	OR	[95% CI]	P-value
Female gender		0.97	[0.24–3.89]	0.965	1.25	[0.32–4.89]	0.746
Age	per year	1	[0.95–1.06]	0.939	0.98	[0.93–1.04]	0.568
Types of diabetes	II vs. I	1.27	[0.32–5.07]	0.74	3.21	[0.77–13.37]	0.109
Degree of haemorrhage				0.592			0.497
	1 vs. 0	0.7	[0.08–6.25]		0.65	[0.07–6.29]	
	2 vs. 0	2.79	[0.48–16.26]		1	[0.19–5.16]	
	3 vs. 0	1.64	[0.22–12.01]		0.21	[0.02–1.81]	
Degree of neovascularization							0.233
	0 vs. 2				0.39	[0.02–8.61]	
	1 vs. 2				3.18	[0.70–14.53]	
Degree of fibrosis				0.61			
	2 vs. 1	1.84	[0.10–33.09]				
	3 vs. 1	0.88	[0.05–16.15]				
CTGF	per unit increase	1.05	[0.97–1.15]	0.235	1.14	[1.03–1.27]	**0.014**
VEGF	per 10 fold increase	94.3	[3.92 – Inf]	**0.005**	0.33	[0.05–2.39]	0.27
Ratio CTGF/log_10_(VEGF)	per unit increase	1.19	[0.86–1.65]	0.283	2.05	[1.18–3.54]	**0.01**

In a multifactorial model with both CTGF and log_10_(VEGF) levels as predictors for degree of fibrosis, both CTGF (p = 0.008) and log_10_(VEGF) (p = 0.033) were associated with degree of fibrosis: CTGF associated *positively* with an odds ratio of 1.19 (95% CI: 1.05–1.36) whereas log_10_(VEGF) levels showed a *negative* association with an odds ratio of 0.06 (95% CI: 0.01–0.80). These findings explain the strong association between CTGF/log_10_(VEGF) ratios and fibrosis in the univariate analysis.

### Clinical course of proliferative retinopathy after laser or anti-VEGF treatment

**Figure 3:**
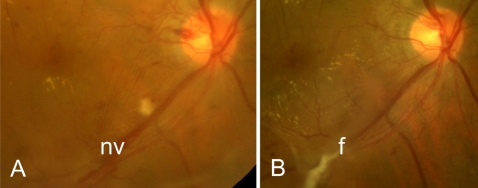
Fundus photographs of a patient with proliferative diabetic retinopathy and new vessels (nv) along the lower vascular arcade, before (A) and 8 months after (B) an injection with bevacizumab followed by pan-retinal photocoagulation. Note the increase in fibrosis (f) after anti-VEGF and laser treatment (B).

**Figure 4:**
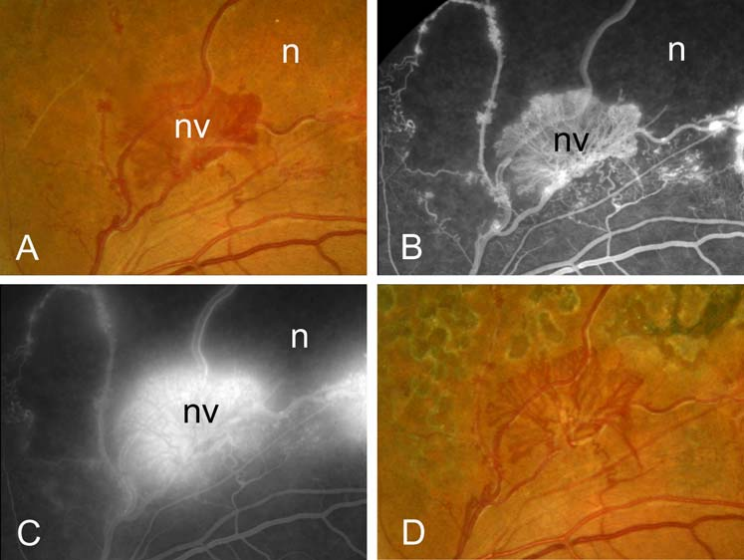
Fundus photographs (A, D) and fluorescein angiographic imaging (B, C) of a patient with branch retinal vein occlusion before (A–C) and after (D) treatment with pan-retinal photocoagulation. Note the leaky vessels consistent with angiogenesis(A, C), and the quiet aspect of the vessels after treatment without formation of fibrosis (D). n, normal; nv, neovascularization.

In each of the 4 cases of PDR, we observed a transition from pre-retinal angiogenesis to a temporary phase of progressing pre-retinal fibrosis after pan-retinal laser treatment, or after anti-VEGF treatment followed by pan-retinal laser (Figure 3). In the case of branch retinal vein occlusion complicated by local pre-retinal angiogenesis with leaky vessels due to ischemia, the new vessels stopped growing and did not leak anymore after laser treatment, whereas fibrosis did not follow (Figure 4).

## Discussion

The observations presented in this study, set against the background of existing literature on VEGF and CTGF, prompt us to propose a novel concept of the regulation of angiogenesis and fibrosis in ocular disease, and in wound healing in general. In this concept, angiogenesis in the vitreous is driven by VEGF, which amongst other factors upregulates the pro-fibrotic factor CTGF in various cell types in the newly formed neovascular membranes. Increasing levels of CTGF inactivate VEGF by reduction of production and complex formation, and when the equilibrium between these two factors shifts to a certain threshold ratio, the angio-fibrotic switch occurs and fibrosis driven by excess CTGF leads to scarring and blindness. This concept identifies CTGF as a major potential therapeutic target in the treatment of ocular fibrosis, in particular in combination with anti-VEGF agents.

What is the evidence that this concept may be true? We have previously shown that vitreous CTGF levels strongly correlate with degree of fibrosis in vitreo-retinal conditions [10], an observation which is in line with the established role of CTGF in pathological fibrosis in other organs [11]–[13], [27]–[31]. However, exogenous CTGF has been reported to have angiogenic effects in ocular experimental models [21], [22], [24], [32]. The present study shows that, in human PDR, the CTGF levels and the ratio of CTGF over VEGF are strongly associated with the presence and degree of fibrosis, whereas CTGF levels, in contrast to VEGF levels, do not have an association with neovascularization. Moreover, multivariate analysis revealed that degree of fibrosis was best predicted by the ratio of CTGF over VEGF. VEGF levels do correlate significantly with active neovascularization, in agreement with the widely accepted role of VEGF as a major ocular angiogenic factor [7], [8], [26], [33]–[35]. We have previously shown that CTGF expression is induced by AGEs in diabetic rat retinas [36], particularly in pericytes [37]. We also have shown in various animal models using CTGF^+/−^ heterozygous and CTGF^−/−^ homozygous CTGF-deficient mice that diminished levels or even complete absence of CTGF did not affect neovascularization significantly [25]. These findings support our concept as outlined above. Our findings question the applicability of the concept of CTGF-driven angiogenesis to in vivo ocular disease. Rather, the accumulated evidence indicates that CTGF effects are highly context dependent and, in the specific condition of PDR, cause fibrosis rather than angiogenesis. Observations by others in PDR patients after VEGF inhibition by intravitreal injections with bevacizumab [38]–[40] may sustain this assumption. In these patients, a remarkable inhibition of angiogenesis is observed. We like to suggest that elevated CTGF levels, which remain in the vitreous after VEGF inhibition, are not able to maintain the angiogenic response, providing further evidence that CTGF has no pro-angiogenic role in PDR.

PDR and other proliferative retinopathies, which represent important causes of blindness, are caused by widespread retinal ischemia. In these conditions, pre-retinal neovascularization into the vitreous is the first sign that a fibrovascular wound healing reaction will develop in later stages. VEGF produced by the ischemic retina is considered to be the major causal growth factor in the neovascularization process [8]. In later stages of PDR, scarring mechanisms within the fibrovascular membranes become more important, and fibrosis and vitreoretinal traction cause retinal detachment and blindness [1], [3], [4], [9]. The mechanisms causing this transition, the angio-fibrotic switch, have not been elucidated.

Further analysis of human vitreous samples in our study demonstrated a significant correlation between CTGF and log_10_(VEGF) levels. This may relate to the fact that VEGF and CTGF are both direct targets of factors such as glucose and AGEs, inherent to the diabetic condition. It may also reflect that VEGF induces CTGF expression in the eye, as has been demonstrated in retinal endothelial cells in vitro [16], [17] and in the rat retina in vivo [18], [36]. The consequent rise of the ratio of CTGF over VEGF may contribute to the angio-fibrotic shift occurring during progression of PDR: it has been reported that CTGF can influence VEGF availability on different levels, including direct protein-protein binding [19], [20], [41] and degradation of HIF-1a, which is a transcription factor for VEGF [42]. Angiogenesis may thus decline due to inactivation of VEGF by CTGF. Subsequent fibrosis may be induced by excess of unbound CTGF as was found in kidney [15], [43] and skin [30]. An earlier study by Kita et al. [44] did not show a significant correlation between CTGF and VEGF in the vitreous of patients with PDR and proliferative vitreoretinopathy. The reason for the difference with the current results is unknown, but may lie in the fact that we analyzed vitreous of PDR patients only.

Our observations that vitreous VEGF levels were significantly higher in active neovascularization than in quiescent neovascularization, and were negatively associated to degree of fibrosis, support this concept. It may, however, not be ruled out that other factors such as fibronectin, integrins and proteoglycans have an influence on the bioavailability of CTGF as well [45]–[47]. Furthermore, several growth factors have been suggested to play a role in angiogenesis and fibrosis in ocular disease [5], [6], which may also have influence on the presence and availability of VEGF and/or CTGF.

Our findings suggest that the balance between VEGF and CTGF determines the angio-fibrotic switch. This concept predicts that a sharp decline of VEGF levels in a patient with active neovascularization due to PDR inhibits angiogenesis, cause the angio-fibrotic switch and temporarily increase fibrosis. In a non-systematic survey, we studied the clinical files of a small series of four PDR patients with active neovascularization treated with intravitreal bevacizumab, an anti-VEGF antibody, and/or pan-retinal laser. Pan-retinal photocoagulation is a therapy which destroys large areas of retina, and which is known to markedly reduce intra-ocular VEGF levels [26]. In these four patients, we did indeed observe regression of neovascularization and the predicted temporary increase in fibrosis. In another patient with branch retinal vein occlusion complicated by focal pre-retinal neovascularization, fibrosis did not follow laser treatment. This suggests that for the angio-fibrotic switch to occur, CTGF levels need to reach a threshold value and a sufficient number of target cells must be able to elicit the fibrotic response. Therefore, intra-ocular fibrosis appears to be a tightly regulated on-off response, like intra-ocular angiogenesis.

Existing literature supports our general concept. The role of CTGF as pro-fibrotic factor is well-established in various organs such as skin and kidney [11]–[13], [15], [27]–[31], [43], [48]–[50] and recently in the retina as well [10], [51]. Furthermore, mRNA and protein of CTGF is upregulated by VEGF in retinal endothelial cells [16]–[18] and co-localizes with VEGF in neovascular subretinal membranes of patients with age-related macular disease [23]. In these membranes, CTGF is localized in retinal pigment epithelial cells [23], which are capable of transdifferentiation into myofibroblasts, the major cell type driving fibrosis. In fibrovascular membranes of patients with PDR and in human diabetic retina, CTGF is localized in myofibroblasts [24] and pericytes [37] which can also transform into myofibroblasts. Finally, in a recent study, it was not only shown that activated human hyalocytes and Müller cells produce CTGF, but also that CTGF does not induce pro-angiogenic functions in cultured human endothelial cells [44].

In summary, our results suggest that, in contrast to VEGF, elevated CTGF levels do not significantly contribute to ocular angiogenesis in PDR. In addition, we propose that CTGF, in a critical balance with VEGF, drives the angio-fibrotic switch and subsequent fibrosis in PDR. This indicates that CTGF-targeted therapy is a possible novel option to prevent sight-threatening fibrosis in PDR and other ocular diseases that are associated with neovascularization and fibrosis. Our concept of the regulation of the angio-fibrotic switch may also apply to wound healing processes outside the eye.
